# Talking therapy: The allopathic nihilation of homoeopathy through conceptual translation and a new medical language

**DOI:** 10.1177/0952695120967872

**Published:** 2021-03-15

**Authors:** Lyn Brierley-Jones

**Affiliations:** University of Leeds, UK

**Keywords:** bacteriology, historiography, homoeopathy, nihilation, symbolic universe

## Abstract

The 19th century saw the development of an eclectic medical marketplace in both the United Kingdom and the United States, with mesmerists, herbalists and hydrotherapists amongst the plethora of medical ‘sectarians’ offering mainstream (or ‘allopathic’) medicine stiff competition. Foremost amongst these competitors were homoeopaths, a group of practitioners who followed Samuel Hahnemann (1982[1810]) in prescribing highly dilute doses of single-drug substances at infrequent intervals according to the ‘law of similars’ (like cures like). The theoretical sophistication of homoeopathy, compared to other medical sectarian systems, alongside its institutional growth after the mid-19th-century cholera epidemics, led to homoeopathy presenting a challenge to allopathy that the latter could not ignore. Whilst the subsequent decline of homoeopathy at the beginning of the 20th century was the result of multiple factors, including developments within medical education, the Progressive movement, and wider socio-economic changes, this article focuses on allopathy’s response to homoeopathy’s conceptual challenge. Using the theoretical framework of Berger and Luckmann (1991[1966]) and taking a Tory historiographical approach (Fuller, 2002) to recover more fully 19th-century homoeopathic knowledge, this article demonstrates how increasingly sophisticated ‘nihilative’ strategies were ultimately successful in neutralising homoeopathy and that homoeopaths were defeated by allopaths (rather than disproven) at the conceptual level. In this process, the therapeutic use of ‘nosodes’ (live disease products) and the language of bacteriology were pivotal. For their part, homoeopaths failed to mount a counter-attack against allopaths with an explanatory framework available to them.

## Introduction

The 19th century saw the development of an eclectic medical marketplace in both the United Kingdom and the United States, with mesmerists, herbalists, and hydrotherapists amongst the plethora of medical ‘sectarians’ offering mainstream (or ‘allopathic’) medicine stiff competition (Rothstein, 1985[1972]). Foremost amongst these competitors were homoeopaths, a group of practitioners following Samuel Hahnemann (1982[1810]) in prescribing highly dilute doses (the minimum dose needed to cure) of single-drug substances (only one drug at a time) at infrequent intervals (often weeks or months apart) according to the ‘law of similars’, or *similia*. This article charts the theoretical and conceptual conflict that ensued between homoeopaths and allopaths throughout the second half of the 19th century and demonstrates how allopathy successfully defeated homoeopathy at the conceptual level. Using Berger and Luckmann’s (1991[1966]) notion of ‘symbolic universes of meaning’ and tuberculosis and ‘nosodes’ (live disease products) as case studies, this article demonstrates how increasingly sophisticated allopathic strategies were ultimately successful in neutralising, or ‘nihilating’, the threat of homoeopathy. This enabled the ‘translation’ of homoeopathy’s core tenets into allopathic knowledge through the language of bacteriology. This article also adopts a Tory historiographical perspective, one that advocates the non-linearity of history (Brush, 1995; Fuller, 2002; Hegel, 1991[1822]) and the potential value of abandoned historical trajectories. As the ‘losers’ of history, homoeopaths lack influence over its telling and struggle to get their own historical voice heard. This article accordingly prioritises the homoeopathic archive and the account therein and uses the terms *allopathy* and *homoeopathy* for parity.

## Historical background

Though the concept was implicit in previous works, Hahnemann was the first to systematise s*imilia similibus curentur* (Handley, 1997), a ‘law’ that specified that when a drug could cause symptoms in a healthy person that same drug could cure a person with a disease producing those symptoms. This was because highly dilute drugs acted upon the vital force, or v*is medicatrix naturae*, the healing power of nature or the ‘spirit’-like force animating the body, an ancient medical principle that had fallen into disuse amongst physicians as it was deemed unreliable and incapable of demonstration (Friedrich Hoffmann, *Medicinae Rationalis*, 1718, quoted in Coulter, 1973: 47).

Hahnemann also coined the term ‘allopathic’, meaning ‘different from symptoms’ or ‘different from suffering’, to denote what he saw as the lack of relationship between drug prescription and symptoms in the practice of his colleagues – or the wrong relationship (*contraria contrariis*: the use of drugs that elicited *opposite* symptoms to those of the disease). This approach, Hahnemann argued, removed symptoms without being curative. Both the relativising of medical practices implied by the term *allopath* and the pejorative overtones it acquired meant that the term was avoided by mainstream physicians in describing themselves.

After reported success in the treatment of cholera in the mid 19th century, homoeopathy gained institutionally and epistemologically, acquiring elite patronage in both countries (Bradford, 1900). In the UK London, Glasgow, and Edinburgh became centres of homoeopathic activity, so that from 1849 London had its own homoeopathic hospital (Nicholls, 1988; Squires, 1985); Glasgow opened its first homoeopathic dispensary, becoming the focus of homoeopathic activity in Scotland for 30 years (Morrell, 1999); and the University of Edinburgh was home to the debate between the homoeopath William Henderson, Professor of Medicine and General Pathology, and James Simpson, Professor of Medicine and Midwifery (Nicholls, 1988). British homoeopaths succeeded in defeating an attempt to make homoeopathic practice illegal under the 1858 Medical Act, enabling the London Homoeopathic Hospital (LHH) to survive.^1^

In the US, homoeopathy’s influence was greater. The American Institute of Homoeopathy (AIH) became America’s first national medical society in 1844, predating the American Medical Association (AMA) by three years. By 1898, homoeopaths in America had 9 national, 33 state, and 85 local medical societies, 39 other local organisations, 57 dispensaries, 20 medical colleges, and 31 medical journals. By the same year, the number of US homoeopaths had risen from 2962 in 1871 to 10,000 (Rothstein, 1985[1972]), giving a ratio of allopaths to homoeopaths of 9:1. Homoeopaths operated 66 general and 74 speciality hospitals, whilst the hospitals of the Homoeopathic Medical College of New York and the New York Homoeopathic College for Women gave students, by 1907, access to 1500 beds – more than the city’s colleges combined. University-affiliated homoeopathic medical schools existed in Boston, California, Iowa, Minnesota, Nebraska, and Ohio, and homoeopaths had developed specialisms in materia medica, ophthalmology, obstetrics, gynaecology, paediatrics, psychology, otology, laryngology, public health, medical ‘sciences’, and surgery (Rothstein, 1985[1972]).^2^ Indeed, in 1876 the head of surgery at the Homoeopathic Medical College of New York, Dr William Tod Helmuth, performed one of the first antiseptic operations in the United States – an ovariotomy – enabling the AIH to report favourably on the practice of antiseptic surgery (ibid.: 259). American homoeopathic schools were amongst the wealthiest in the country, with three of the four largest medical libraries in 1900 existing in homoeopathic medical schools, and two having the greatest material assets in terms of buildings and grounds (Coulter, 1973).

Homoeopaths were also active in public health. In 1873, the homoeopath Tullio Verdi was appointed as health officer to the D.C. Board of Health. Verdi investigated the sanitary laws of several European cities, and at home he established dispensaries and enforced smallpox vaccination laws. Congress’ satisfaction with homoeopathic performance in the yellow fever epidemic of 1878 led to Verdi’s being appointed to the (short-lived) National Board of Health in April 1879. Homoeopathic surgeon generals were further appointed in Rhode Island and New York.

By the early 20th century, however, homoeopathy was in decline in both countries. In the wake of the Flexner report of 1910 in the US, homoeopathic medical colleges closed or became allopathic, with 22 homoeopathic colleges in 1900 becoming 7 by 1918 (Brown, 1979). At the same time, allopathic medical societies, including the AMA, reopened their doors to homoeopaths (Coulter, 1973; Rothstein, 1985[1972]).^3^ In the UK, the *British Journal of Homoeopathy* ceased publication in 1884, and the number of registered homoeopaths fell from 299 in 1874 to 201 in 1909.

Furthermore, the advent of philanthropic and centralised funding of medical education (Brown, 1979) against a backdrop of Progressivism (Hofstadter, 1962) in the US and, later in Britain, of state-managed medical provision culminating in the formation of the National Health Service in 1948, crowded homoeopathy out, by degrees, from medical curricula and other key areas (Nicholls, 1988; Rothstein, 1985[1972]). Homoeopathy’s decline in Britain may have been less dramatic than that in the US (Chou, 2016), but it was equally damaging to the profession given the theoretical and clinical influence of the likes of James Compton-Burnett in both countries (Nicholls, 1988).

## Theoretical framework: Berger and Luckmann’s divergent symbolic universes and machineries of universe maintenance

Berger and Luckmann (1991[1966]: 122–5) argue that once knowledge becomes divorced from direct experience, ongoing legitimation of that knowledge is required. Various levels of legitimation of knowledge exist, but the most sophisticated is the ‘symbolic universe’: a body of theoretical tradition that integrates different provinces of meaning and encompasses the institutional order into a symbolic totality. Legitimation of such a universe requires ‘machineries of universe maintenance’ (ibid.: 122). A perfect social system would be self-maintaining, but systems are rarely so closed and efficient in their socialisation processes. Universe maintenance becomes especially necessary when a deviant conception of reality threatens the maintenance of the symbolic universe and the institutional order.

At the conceptual and symbolic level of status quo maintenance, ‘nihilation’ represents a strategy capable of neutralising an external threat. Nihilation can occur at the first or second order. The first-order strategy of rejection and denial is straightforward: the phenomenon threatening one’s world view (e.g. cured homoeopathic patients) is denied any ontological status. It is not real. The second-order strategy is more complex and seeks to explain the knowledge and practices of the deviant knowledge system in terms of one’s own conceptual machinery so that the knowledge is ‘neutralised’ or ‘liquidated’. In this process of translation, the knowledge base of the receiving symbolic universe can be modified, or even changed radically. Knowledge is successfully nihilated when a loss of cognitive authority of the rival group occurs. As Berger and Luckmann (1991[1966]: 133–4) put it,

The presupposition is always that the negator does not really know what he is saying. His statements become meaningful only as they are translated into more ‘correct’ terms, that is, terms deriving from the universe he negates.…If the symbolic universe is to comprehend all reality, nothing can be allowed to remain outside its conceptual scope.

A social group and its knowledge base may survive a nihilistic attack from a dominant or competing group, but nihilation reduces a group’s cognitive distinctiveness and thus its identity. Allopathic and homoeopathic medicine can be seen as representing different symbolic universes, both of which required maintenance, but allopathy particularly once it came under threat from homoeopathy.

## Historiography and the construction of difference

As well as using the framework of Berger and Luckmann, this article takes a Tory historiographical perspective (Fuller, 2002). As a result, it seeks to go beyond placing homoeopathy in a broader sociocultural context, crediting its practitioners and patrons with a certain rationality, or merely understanding homoeopathy in its own terms (Gevitz, 1988); an ostensibly relativist and Prig position (Fuller, 2002). It also rejects the position that we live in the best of all possible worlds (Alvargonzález, 2013; Butterfield, 1931); that homoeopathy’s demise was inevitable (Haller, 2014 and Whorton, 2002 are notable exceptions here) or the result of the advent of scientific medicine (Chou, 2016; Rothstein, 1985[1972]); or, indeed, the ultimately Whiggish position that *all* medical history is the history of the placebo (Shapiro and Shapiro, 1997).

The Tory stance requires a fuller recovery of the lost knowledge (*prisca sapienta*) associated with an abandoned trajectory and a ‘suspension’ of the received view, a different focus within the archive, and an imagining of historical events as having the potential to turn out differently. This, however, presents a challenge. Not only does history tend to be written by the winners, of which the Tory historian has to be mindful (Brush, 1995; Fuller 2002), but the historical archive is composed of ‘what leaves an impression’, of what is deemed worth keeping (Fuller, 2002: 399). That is, the archive *in toto* does not represent equally all historical players. To redress such an imbalance, and to return the repressed more fully to consciousness, this article investigates homoeopathy extensively from within the homoeopathic archive, particularly through homoeopathic journals and medical society transactions associated with ‘mainstream’ homoeopaths in both countries; that is, the AIH in the US and the British Homoeopathic Society in Britain.^4^ These sources document some of the scientific and experimental activities of homoeopaths: their new discoveries, arguments, and defences. This is not to suggest that these sources offer the complete picture of homoeopathy; indeed, they show particular facets of homoeopathy, albeit important ones. Nor is it to deny variation in practice amongst homoeopaths or the value of other historical sources. But given that homoeopathy represents a losing side, the sources used here enable us to see homoeopathy from inside ‘the camp’ (Bloor, 1978) and to understand what homoeopaths sought to achieve. These are also sources that are available for practitioners in both countries. The argument made here is that whilst homoeopathy is not completely lost, as it continues to the present day, the ‘scientific’ programme of homoeopathic research that began in the 19th century and was centred within many of these institutions *is* lost.

## Divergent symbolic universes

### Homoeopathic and allopathic metaphysical differences at the beginning of the 19th century

A major point of departure at the beginning of the 19th century between allopaths and homoeopaths was the *vis medicatrix naturae*, or ‘healing power of nature’. The heroic system of the former derived from the solidist teachings of the Scottish physicians William Cullen (1710–90) and John Brown (1735–88), as well as the American physician Benjamin Rush (1745–1813). Based upon theoretical analogies with mechanical, chemical, and hydraulic processes, heroic medicine was characterised by interventions to control disease by overpowering it. Homoeopaths, by contrast, claimed that cure could be achieved only by stimulating the body’s natural ability to heal itself, as it was the body’s own powers of self-repair that succeeded in curing, not the medicine. The *vis medicatrix naturae* was a key concept for homoeopaths and underpinned their practice. With the dominance of *contraria contrariis* within allopathic heroic medicine, characterising particularly the first half of the 19th century, the concept of *vis medicatrix naturae* fell into disrepute and was largely abandoned by allopaths (Haller, 2009). Only a younger minority of the allopathic profession entertained the notion of *vis medicatrix naturae*, and that was mainly theoretical (Whorton, 2002). Benjamin Rush, for example, chided physicians in 1811 for ‘an undue reliance on the powers of nature in curing disease’, as ‘the principle is devoid not only of intelligence, but possesses no healing power of any kind’ (Coulter, 1973: 49). Likewise, Nathaniel Chapman (1780–1853), successor to Rush’s chair in materia medica at the University of Pennsylvania, commented in 1816 that the idea that ‘fever will run its course and that all practitioners can do is abate its force is a dangerous one and should be combatted.…It begets a feeble practice and suffers the disease to go on til it is beyond our power’. In 1830, Chapman reiterated the feelings of insecurity that the *vis medicatrix naturae* evoked in some physicians when he said, ‘Could I believe this opinion [the vital force] to be correct, I would at once without hesitation strike the flag of my profession, and cease to pilfer a generous public of their money by such a fraud and impostance’ (quoted in Warner, 1997: 18–19). By contrast, Hahnemann stated that in disease, ‘medicinal substances capable of acting on the organism exert their non material (dynamic) influence only on the spirit like vital force’ (Hahnemann, 1982[1810]: 15). However, Hahnemann agreed with both Rush and Chapman that the vital force should not be left to ‘act of itself’, was devoid of intelligence, and required direction (ibid.: 25).

### Allopaths’ first nihilative attempt: Denial and rejection

These two alternative medical metaphysics were put to the test from the 1830s onwards, when Europe and America experienced successive cholera epidemics. Hahnemann determined that the principal drugs for cholera’s three stages were *camfora* (camphor), *cuprum* (copper), and *veratrum* (white hellebore). Frederick F. H. Quin (1799–1878), who introduced homoeopathy to Britain, reported a mortality rate of only 5% in 500 cases of cholera in Moravia, a location where allopaths reported a 50% mortality rate. In Russia, homoeopaths reported a mortality rate of 21.1% in the 1832 epidemic, whilst allopaths reported 74.19%. Even no treatment was reportedly superior to allopathy in cholera, the former producing a mortality rate in Russia of 67.34%. In Vienna, homoeopaths reported a mortality rate of 8% and allopaths 31%, whilst the LHH in 1854 reported such impressive mortality figures to Parliament (16.4% for homoeopaths compared to 77% for allopaths) that the returns of the hospital were suppressed, ultimately unsuccessfully (Squires, 1985: 381). In 1900, the homoeopathic historian T. L. Bradford (1847–1918) reported that the aggregate statistics of treatment results for cholera in Europe and America were 40% for allopathic treatment and 9% for homoeopathic treatment.

The initial allopathic response to these figures was denial, followed by rejection. Allopaths claimed that cholera patients cured by means of homoeopathy were either fictitious or not real cholera cases, but instead suffered from general gastrointestinal disturbances (Squires, 1985). In response to such accusations, the LHH requested independent inspection in 1854, to which the Medical Council eventually agreed. Its emissary, Dr MacLoughlin, was able to confirm both that the LHH was dealing with genuine cases of cholera and that the hospital was at the very heart of the epidemic in St James’, Westminster. Similarly, in the US, two Cincinnati-based homoeopaths responded by publishing in newspapers their loss of 35 patients to cholera out of 1116 treated in the 1849 epidemic, along with the names and addresses of all patients (Coulter, 1973).

Denial alone thus proved ineffective, and with the inauguration of national allopathic medical societies in America and Britain (the AMA and the British Medical Association) in 1847 and 1850, respectively, many American medical societies began to openly reject homoeopaths. A ‘consultation clause’ was incorporated into the codes of ethics of many American societies, and a range of exclusionary tactics were adopted in the UK. The objective was to restrict, or ban altogether, the participation of homoeopaths (and other ‘medical sectarians’) in allopathic medical societies, as well as medical referral to, and later any professional or personal dialogue with, homoeopaths (Coulter, 1973; Nicholls, 1988; Squires, 1985). This institutional division and stigmatisation was, in part, intended to prevent more allopaths defecting to homoeopathy (most homoeopaths by mid-century had ‘converted’ from allopathic medicine), but this arguably backfired and strengthened homoeopathy further (Coulter, 1973).

### Sophisticated secondary nihilative strategies: Reincorporating the healing power of nature

Homoeopathy’s comparative theoretical sophistication, institutional development, and reported therapeutic efficacy combined to make it resistant to the simpler nihilative techniques used effectively on other deviant medical systems (Forbes, 1846). The failure of denial and rejection meant a secondary, more sophisticated nihilative move was required. Here, allopaths argued that both the failure of their own treatment and the apparent successes of homoeopathy were explicable by the same phenomenon: the body’s natural healing powers. Homoeopaths cured cholera because, unlike allopaths, they were allowing the body to heal itself, since prescribing the dilute doses used in homoeopathy was tantamount to doing nothing at all. Allopaths, on the other hand, were preventing the operation of this principle by giving medicine, principally depletives, when medicine was not needed. Hence, the *vis medicatrix naturae*, along with the concept of self-limiting diseases, gained greater acceptance in allopathic circles (Coulter, 1973).

John Forbes was one of the first to make such a secondary nihilative move. In his *Homoeopathy, Allopathy and Young Physic* (1846), Forbes made three points about homoeopathy: one, that homoeopathic remedies were medicinally inactive; two, that patients *did* recover under homoeopathic treatment; and three, that, therefore, the excessive drugging and depletion characteristic of allopathic practice was unnecessary (Nicholls, 1988). Indeed, Forbes was particularly nihilative when he reinterpreted Hahnemann’s original clinical observations as mistaken and homoeopathic dilutions as ‘seemingly beneficial’ (Forbes, 1846: 7–8). Forbes pointed out why such a response to homoeopathy was necessary, admitting that if homoeopathy had descended on the world as only a theory, then it would have posed no threat to allopathy. Instead, homoeopathy arrived

as a conqueror, powerful, famous, and triumphant. The disciples of Hahnemann are spread over the whole civilized world. There is not a town of any considerable size in Germany, France, Italy, England or America, that does not boast of possessing one or more homoeopathic physicians, not a few of whom are men of high respectability and learning: many of them in large practice, and patronized especially by persons of high rank. (ibid.: 77)

Thus, by 1860, Oliver Wendell Holmes (1809–94), a Harvard medical graduate and prominent medical spokesman who served as dean of Dartmouth Medical School, could address the Massachussetts Medical Society on the *virtues* of the healing power of nature (Holmes, 1860). Though Holmes’ address was initially publicly disowned by the Society, as a leader of the profession Holmes could not be ignored (Coulter, 1973). By 1873, the AMA’s president, Thomas M. Logan (1840–1914), alluded to allopathic belief in the vital force, claiming that ‘science…in her researches after truth has found that a large number of acute diseases, occurring in previously sound persons, have a tendency to terminate in the restoration of health even though no drug has been given’ (Logan, 1873: 81).

To be sure, homoeopathy was not the sole influence on allopathy and heroic medicine. Expectant therapy from Paris, which emphasised moderate, gentle therapeutics and less drugging, began to make inroads into American medicine through the work of Alfred Stille (1813–1900), William Gerhard (1809–72), and Samuel Morton (1799–1851), graduates of the Philadelphia Hospital and Medical College who studied in Paris and used Parisian methods in researching consumption, typhoid, and typhus (Porter, 1997). In Britain, Parisian methods were introduced through Thomas Hodgkin (1798–1866), a stethoscopist and lecturer at Guy’s Hospital who learned directly from Laennec; Thomas Addison (1793–1860), who later discovered ‘pernicious anaemia’ and Addison’s disease; and Addison’s colleague Richard Bright (1789–1858), who established an effective diagnostic test for the kidney condition that came to bear his name, also used Parisian methods (ibid.). Through their association with Parisian medicine, these men, and the papers and monographs emerging from the Continent, led to wider adoption of the *medecine expectante*. But it was sceptical empiricism as an approach, rather than any specific therapeutic measures, that had permeated American medicine by mid-century (Warner, 1997). Indeed, Porter argues that therapeutic nihilism, whilst perfectly adapted to the French charity hospital, proved ‘hopeless for a nation of intrepid pioneers’ such as America, and that heroic drugging actually emerged as a stand *against* French therapeutics (Porter, 1997: 319). Thus, restoration of the *vis medicatrix naturae* was facilitated primarily by its native American roots, its Paracelsian ancestry, and heroic medicine’s declining popularity with the public (Coulter, 1973; Warner, 1997). Into this mix stepped homoeopathy, the reported successes of which could be naturally ‘explained away’ by the therapeutic nihilism, vital force, and doctrine of self-limiting disease associated with the Parisian school. As the AMA president of 1873, Logan, argued,

Accumulated observations have established the fact that certain acute diseases run a definite course and end spontaneously at a certain period from their onset. Conclusions, therefore drawn…as to the efficacy of drugs to cut short their duration, are thus proved to be founded on false premises, and consequently are not trustworthy. (Logan, 1873: 82)

Demonstrating that he particularly had the ‘false premises’ of homoeopathy in mind, the president concluded, ‘It is precisely on such garbled interpretations of what science has ascertained, that empirics, mingling a crude smattering of knowledge with a cloudy mass of ignorance, have erected their crazy structures of *infinitesimal* nonsense’ (ibid.; emphasis added).

Hence, allopaths accused homoeopaths, and the public, of making ‘faulty interpretations’. The only legitimate arbiter of *all* medical data was the ‘scientific physician’ – in other words, the allopath, who alone was capable of rejecting ‘the hostility conceived and immature speculations of the self-satisfied empirics’, whilst simultaneously engaged in ‘the judicious employment of the rational means at his command…pure air, food and stimulants included, [to save] the patient from death’ (Logan, 1873: 83).

As Berger and Luckmann argue, nihilation strategies as techniques of (de)legitimation have the effect of transforming the symbolic universe responsible for the nihilating manoevre. In this instance, allopaths increasingly came to acknowledge the role of the vital force in healing (Coulter, 1973; Warner, 1997) in terms of its being a *benevolent* principle. This was in contrast to Hahnemann, who conceived of the vital force as being a dumb, brutish thing, which in illness required constant direction, it alone being insufficient to restore health: ‘The vital force was given to us to sustain our life in harmony as long as we are healthy, not to heal itself when diseased, for if it possessed an ability so worthy of imitation it would never allow the organism to fall ill’ (Hahnemann, 1982[1810]: 25). Indeed, Hahnemann even considered the vital force to be the instrument of death, claiming, ‘If such help is not forthcoming, it [the vital force] tries to save itself at all costs by increasing the suffering and especially by violent evacuations, often at the cost of tremendous sacrifice, sometimes at the cost of life itself’ (ibid.). Hence, Hahnemann’s concept of the vital force only minimally embraced the idea of self-limiting diseases and differed from the allopathic variant.

### The incorporation of small doses into allopathic practice: The case of tuberculosis and homoeopathic nosodes

Further translations of homoeopathy ensued in both medical theory and practice. The minimum dose, a key homoeopathic tenet that held that only minute amounts of a drug were required to stimulate a reaction and cure, made its way into allopathic practice especially via serum therapy and attained particular therapeutic heights in the treatment of tuberculosis. J. M. Sims (1813–83) instructed the AMA meeting of 1875 on using small drug doses, recommending clinical experience as the guide (Sims, 1875). By the turn of the century, the small drug doses once lampooned by allopaths were central to their practice. Almroth E. Wright (1861–1947), Director of the Institute of Pathology at St Mary’s Hospital, London, had demonstrated that the smallest dose of a specific vaccine was able to stimulate antibody production (Wright, 1907). Subsequently, Nathan Raw noted that initial doses of tuberculin should start at 1000^th^ or 2000^th^ of a milligram in treating early tuberculosis, with doses rising rapidly thereafter until a ‘marked reaction’ was produced (Raw, 1910: 845) and Philippi used ‘extreme individualization’ in using tuberculin to lower the fever of tuberculosis patients with doses ranging from 3-6 millionths of a milligram (Current Medical Literature, 1910a: 442).

Likewise, in 1910, R. W. Philip, allopathic physician to the Royal Infirmary, Edinburgh, recommended small doses of tuberculin in treating tuberculosis, stating, ‘It is best to begin treatment with small dosage…and by gradual increase, if no effect has been produced, it is commonly easy to determine the *minimal dose* which is *effective*’ (Philip, 1910: 21; emphasis in original). How small was small? Philip recommended 0.0001 g of Koch’s original tuberculin as an initial dose, or 1/5000 to 1/2000 mg of Koch’s TR, or 0.1 cm^3^ of a 1 in 100,000 solution of Beraneck’s tuberculin. The 1 in 100,000 solution of Beraneck’s tuberculin corresponded to 10^–5^, which approximates to midway between 2C and 3C on the homoeopathic centesimal scale. For homoeopaths, serum therapy and Wright’s opsonic index represented a measure of the action of the vital force and further validated homoeopathic practice.

Indeed, the AIH viewed Wright and other bacteriologists as homoeopaths in disguise. Wright himself acknowledged, ‘This is pure homoeopathy’, and even the renowned immunologist and Nobel Prize winner Emil von Behring (1854–1917), whilst working on a new tuberculo-therapeutic substance, conceded,

[Tuberculin’s] therapeutic usefulness must be traced in origin to a principle which cannot be better characterized than by Hahnemann’s word ‘homoeopathic’. What else causes immunity in sheep vaccinated against anthrax, than the influence previously exerted by the virus, *similar* in character to that of the fatal anthrax virus. And by what technical term could we more appropriately speak of this influence exerted by a *similar* virus than by Hahnemann’s word, ‘homoeopathy’? (Linn, 1907: 317; emphasis in original)

Making explicit the allopathic use of homoeopathic dilutions, the homoeopath W. H. Watters pointed out that the allopathic recommended first dose of tuberculin of 0.000001 g corresponded to the homoeopathic 6X, whilst the less cautious but often more satisfactory doses of 0.0000001 to 0.00000001 g corresponded to the homoeopathic 7X and 8X dilutions, respectively (Watters, 1907).

Not only did allopaths come to use small doses according to *similia*, but they began to individualise the dosage just as homoeopaths always had. The JAMA of 22 January 1910 noted, ‘The [tuberculin] dosage is at present empirical; each individual case must be an experiment, and the symptoms carefully observed after each dose’ (Baldwin, 1910: 261). Edward Baldwin, a New York–based allopathic physician, reminded readers that in treating tuberculosis with tuberculin, ‘clinical oversight is the most satisfactory guide’ (ibid.: 261–1), with ‘individual cases requiring individual treatment’ (Maguire, 1900: 1695). Allopaths even began to incorporate the mental and emotional symptoms of tuberculosis into their conceptualisation of the disease, with the JAMA referring to the ‘psychopathology’ of the tuberculous patient and the ‘tuberculous personality’ having a ‘peculiar egotistic, irritable, spoiled child attitude…entirely contrary to what the same individuals presented in health’ (Current Medical Literature, 1910b: 725).

Thus, by the turn of the 20th century, allopaths had reintroduced the *vis medicatrix naturae* into their practice, and translated the once pilloried *similia*, minimum dose, and mental aspect of disease into their own universe of meaning. With such similar (but not identical) therapeutic practices, how were allopaths to maintain their distinctiveness from homoeopathy and resist the stigma associated with it? With conceptual synthesis, how were they to preserve their medical identity? A contributing factor was the construction of difference and a separate allopathic identity, legitimated initially through the therapeutic nihilism of the Paris School and later, more comprehensively, through the language of bacteriology.

## The management of ‘nihilation’ through language

### From ‘*similia*’ to ‘vaccine’; from ‘triturating’ to ‘pulverising’

The translation of homoeopathic concepts into the allopathic symbolic universe is particularly evident in the development and use of the substance tuberculin. The use of such live disease products, ‘nosodes’, in treating tuberculosis originated with James Compton-Burnett (1840–1901), a British homoeopath with a large London practice and a physician at the London Homoeopathic Hospital. Burnett was the first to experiment with the live tubercle bacillus, initially in secret, between 1875 and 1883 (Burnett, 1894). Burnett reported acquiring diseased lung tissue from a local hospital pathologist.^5^ Processing this material according to the homoeopathic method, Burnett experimented on over 50 tuberculous patients, publishing his results only after it was professionally safe to do so – that is, after Koch (1843–1910) had revealed his own discovery (Burnett, 1892). Both Koch and Burnett admitted with time that their respective preparations (Koch-tuberculin and Burnett-bacillinum) contained the toxin of tuberculosis (Burnett, 1892, 1894; Gradmann, 2009). Knowledge of the therapeutic failure of Koch’s preparation soon became widespread and threatened his reputation, whilst Burnett and later homoeopaths who used tuberculin (Koch’s preparation) more carefully reported multiple successes (The Use of Tuberculin Part III, 1914; Burnett, 1892, 1894; Cooke, 1892; Gradmann, 2009). Initially, homoeopaths used both Koch’s and Burnett’s preparations, whereas allopaths reported using only that of Koch. Later, a wider range of preparations became available (Allen, 1910). In 1910, the allopath Philip claimed that in using tuberculin, ‘we make use of an agent *closely related to the infecting organism*, and there is abundant ground for the belief that we thereby reinforce nature’s own effort at immunisation’ (Philip, 1910: 20; emphasis added). Strictly speaking, of course, this post hoc ‘vaccine’ was prophylactic only as long as treatment continued and was administered to those already suffering from the disease. It was, however, a way of describing the process of administering the similimum without recourse to homoeopathic terminology (ibid.: 261).

Philip also described the ‘aggravation’ that was always taken by homoeopaths as a positive sign a remedy had been well selected. In *The Organon*, Hahnemann (1982[1810]: 129) had stated, ‘The so-called homoeopathic aggravation, or rather the primary action of the homoeopathic medicine, which appears to increase somewhat the symptoms of the original disease takes place in the first hour or in the first few hours’, followed by a reversal of this process. Correspondingly, Philip’s observation was, ‘Immediately following the first injection of a suitable dose [of tuberculin] the gland may be found slightly enlarged and possibly tender. The gland is congested. In the course of a few days the gland under observation will be found reduced in size’ (Philip, 1910: 20).

Edward Baldwin, an allopathic physician, referred in the JAMA in 1910 to tuberculin as a ‘vaccine…composed of the pulverized insoluble substance of the bacillus itself’ (Baldwin, 1910: 260). In the same volume, the JAMA noted that in the New Tuberculin ‘the germs are simply crushed and pulverized and mixed with equal parts of water and glycerine’ (New and Non Official Remedies, 1910: 288). This ‘pulverising’ (Baldwin, 1910: 269) had been practised by homoeopaths according to Hahnemann’s (1982[1810]: 190–6) directions in *The Organon* for over a century in the breaking down of substances before attenuation (dilution). Hahnemann used both ‘trituration’ and ‘pulverization’ to describe this process of drug preparation, even advising the use of a ‘glazed porcelain mortar’ and a ‘porcelain pestle’ (ibid.: 192). Indeed, trituration was central to homoeopathic drug preparation, and there had been much homoeopathic debate in the last quarter of the 19th century about its power to prepare insoluble substances for dilution, especially metals (Wesselhoeft, 1877a, 1877b, 1882). Now allopaths were using this method in preparing tuberculin. Charles Wheeler explained to the British Homoeopathic Congress in 1909 that the allopathic use of tuberculin

comes nearer to homoeopathic practice than their use of other vaccines; firstly because its preparation *breaks up the bodies of the bacilli* in a way that is not done in the making of ordinary vaccines; and secondly, because, following Dr Latham, it is frequently administered by mouth. (Wheeler, 1909: 482; emphasis added)

### Renaming the ‘vital force’ and the ‘minimum dose’

By the turn of the 20th century, allopaths were referring to their doses as the ‘most minute’, ‘small’, and ‘minimal’ (Crowe, 1910: 1130; Current Medical Literature, 1910c: 659); homoeopaths, as the ‘minimum’, ‘dilute’, or ‘infinitesimal’. Allopaths measured their dilutions in terms of a fraction of a milligram (previously a ‘grain’; New and Non Official Remedies, 1910: 288; Wethered, 1910: 987), whereas homoeopaths called these ‘potencies’ and had devised their own scales of measurement: the decimal and centesimal scales, where one part drug was diluted into 10 and 100 parts water or alcohol, respectively (Coulter, 1973).

The homoeopathic ‘aggravation’ became, in the hands of allopaths, the local or general ‘reaction’ – or, in Wright’s vocabulary, ‘the negative phase’ (Baldwin, 1910: 261) – with the reaction being ‘proportionate to the smallness of the dose’ (ibid.: 260). This was followed by improvement, or ‘the positive phase’ (Crowe, 1910: 1132). Stimulating the ‘vital force’ became the practice of ‘stimulat[ing] the *disease focus* to heal’ (Baldwin, 1910: 261; emphasis added) or the ‘natural protective mechanism’ (Philip, 1910: 20). Allopaths still did refer to the ‘vital force’ or ‘powers’, but this phrase was beginning to be replaced with ‘immunity levels’ and ‘resistance’ (Maguire, 1900: 1695), or ‘antigens’ and ‘antibodies’ (Crowe, 1910: 1134). Furthermore, vital capacity was claimed to be measurable. Bandelier reported in the JAMA of 1910 that the ‘vital capacity’ of some patients ‘increased by 290 cc, the excursions by 0.91 cm’, whilst the vital capacity of others increased by 360 cc and the excursions by 1 cm (Bandelier, 1910).

Conceptual translation could go both ways. The Iowan homoeopath A. M. Linn asked whether, in light of Wright’s opsonic hypothesis, homoeopaths would not be justified in substituting for Hahnemann’s concept of ‘psora’ the term ‘lowered opsonic index’ (Linn, 1907: 317). These conceptual equivalences are summarised in Table 1 above.

**Table 1.** Medical synonyms in use by homoeopaths and allopaths in their treatment of tuberculosis around 1910.

Allopaths	Homoeopaths
Natural protective mechanism	Vital force
Immunity	Cure
Vaccine	Similimum
Small/minimal dose	Minimum/infinitesimal dose
Local/systemic disturbance/reaction/negative phase	Aggravation
Patient’s aspect	Subjective symptoms
Solution	Dilution
Dose/name of specific drug	Drug
Pulverised	Triturated

## Reverse drug action and the homoeopathic explanation that never was

In contrast to allopathy’s nihilistic strategies, homoeopaths failed to elaborate allopathic success in terms of their own world view. Indeed, in contrast to that of Hahnemann, homoeopaths’ appeal to homoeopathic theory had diminished *in toto*. The strength of the concept of the ‘reverse action of drugs’ was its universality, and its potential to explain the action of material (low) and dilute (high) drug doses, whilst enabling homoeopaths to claim the superior, curative effect of the latter. Hahnemann (1982[1810]: 57) had explained in *The Organon* the opposite effects of the primary and secondary drug actions in drugs such as *digitalis purpurea*. So when George Ockford, an Indianapolis-based American homoeopath, called for more work to be done on reverse drug action, it might have been a propitious moment in homoeopathic history. However, Ockford’s rationale was to clarify the burgeoning and often contradictory homoeopathic materia medica rather than to explain allopathic effects in homoeopathic terms (Ockford, 1878).

The reverse action of drugs offered homoeopaths the opportunity to mount a nihilating counter-attack against allopaths. It is particularly easy to imagine this possibility when recalling that homoeopaths believed knowledge of pathological and physiological drug action contributed to their drug epistemology and therefore to therapeutics. Even the very high-potency homoeopath James Tyler Kent (1849–1916) acknowledged the utility of post mortem findings in furthering homoeopaths’ understanding of drug action in relation to a drug’s organ and tissue affinities (Kent, ca. 1906). In particular, poisonings were considered useful, as they revealed the destructive tissue changes that a proving, could not be ethically permitted to produce (through overdose and ‘aggravation’). Thus, in discerning the full action of a drug and its influence on body processes *and* tissue structure, post mortem findings were useful for producing a more complete drug picture. In practice, this was believed to increase the likelihood of the homoeopathic physician selecting the correct drug the first time around.

Equally damaging was the fact that when homoeopaths acknowledged their own use of drugs in material doses, they did not explain them in terms of the continuum of drug action.

### Categories rather than continuum

By the turn of the 20th century, homoeopaths had adopted a categorical rather than a continuum-based explanatory framework, and rather than expanding, their symbolic universe was contracting. Eldridge C. Price, an American homoeopath based in Baltimore, Maryland, outlined the respective therapeutic spheres of the ‘four pathies’: antipathy, allopathy, isopathy, and homoeopathy (Price, 1898). Price began by admitting that few homoeopaths were now exclusivists but had outgrown sectarianism, being simply physicians, so that ‘we believe in allopathy, in antipathy, and in Homoeopathy, each in its own place, and with a scientific reason for our beliefs, and we want the world to know it’ (ibid.: 105).

Homoeopathy thus became relegated to a technique or skill set, one of several that the ‘scientific’ physician had at his disposal. The homoeopath W. H. Geohegan (1898: 122) added,

The dominant school of physicians justly repudiate the term ‘allopathic’. The drugs chosen by their methods do not always bear the allopathic relationship; in fact, the use of similars abounds in their practice. This has been openly admitted, of recent years, by some of their leading authorities…[as has the fact that it] was necessary [for homoeopaths] to ascertain the proper limitation of the sphere within which the law of Similia is applicable.

Homoeopaths’ old adversary H. C. Wood, the Pennsylvanian physician and biologist famed for his *Treatise on Therapeutics* (1874), was cited as recognising that *similia similbus curentur* had survived for 2300 years, thus, ‘it must possess some peculiar vitality, some measure of truth, and I myself believe that, as a rule of practice, it will *at times* lead to a good result’ (Geohegan, 1898: 122; emphasis added).

Disastrously, the homoeopath Price asked, ‘What if, in following truth, we are led away from Homoeopathy? It matters not’. Subsuming the law of *similia* under the possibility of greater truths, Price claimed, ‘We will only be drawing nearer to the fact, to the roots of the universe, to that which is the *cause* of the law of similars’ (Price, 1898: 106; emphasis added). Thus, it was believed the homoeopathic law of *similia* would be subsumed under some other, greater law, rather than vice versa. By the end of the century, leaders of the homoeopathic profession were arguing, contra Hahnemann, that homoeopathy was one route to truth and not truth itself.

Benjamin F. Bailey, the AIH president, lamented in 1905 the homoeopathic indifference to explaining the positive clinical outcomes of allopaths and homoeopaths’ over-reliance on outright rejection of any allopathic successes:

Contented in our own sufficiency [he told the Institute] we were unwilling to grant or recognise any accomplishment the result of scientific studies that might have been made by the regular school in the last twenty-five years, and we were inclined to change positions and to cry out against every new discovery – not on account of its negative evidence but on account of its origin. If it came from the regular school it must be false, it must be bad. (Bailey, 1905: 104)

Thus Bailey admitted that homoeopaths had not taken developments and discoveries within allopathy seriously. They had not grappled in detail with allopathic knowledge on a conceptual or theoretical level. Homoeopaths had remained at the first level of nihilation – denial and rejection – assuming that allopathic successes and developments did not merit explanation (Berger and Luckmann, 1991[1966]: 130). Homoeopaths paid dearly for their complacency. ‘We rested on our oars’, Bailey said. It was a loss from which they would not recover.

Recovering homoeopathy’s history more fully through a Tory historiographical perspective reveals the extent of the challenge homoeopathy presented to allopathy at the conceptual level, a challenge that allopathy could not ignore. Far from being a spiritual or anti-materialist medical system, homoeopathy fused highly diluted drugs with knowledge of pathology and physiology. By the end of the 19th century, homoeopathy was at the cutting edge of new therapeutic developments in the treatment of tuberculosis, a fact that was acknowledged at the time. Allopathy met this challenge with a series of progressively escalated nihilation strategies that enabled it to translate the core tenets of homoeopathic philosophy and practice into its own universe of meaning, the language and conceptual framework of bacteriology being the most successful and comprehensive of these strategies. Homoeopathy, on the other hand, was unable to repeat Hahnemann’s earlier success of creating an explanation for allopathy’s successes in its own terms. Analysing the historical record from the loser’s point of view does not reveal the weakness or failure of homoeopathy, but rather shows just how close homoeopathy came to becoming medical science.

## Conclusion

Throughout the 19th century, allopaths were highly effective at absorbing and neutralising conceptual threats to their symbolic universe. As homoeopathy posed a greater and longer-lasting threat than other deviant medical systems, such as herbalism, these neutralising techniques extended beyond simply separating its beliefs and practices from those of the competition (Haller, 2014) or admitting homoeopathic drugs into their own materia medica (Coulter, 1979). After denying ontological status to homoeopathic cures and excluding homoeopaths from their medical societies, allopaths emphasised the self-limiting nature of acute diseases and consequently the power of the *vis medicatrix naturae*. This led to allopaths absorbing a specific form of vitalism into their own practice. Whilst allopaths’ conception of the vital force was modified through translation, as Berger and Luckmann’s theory would suggest, it began to neutralise the homoeopathic threat. The same approach was successfully deployed against the concepts of the law of similars and the minimum dose. Serum therapy in the treatment of tuberculosis, *the* disease of the 19th century, manifests these processes. Allopathy was thus able to use a range of homoeopathic drugs, as well as homoeopathic techniques and theoretical principles, effectively whilst ‘explaining away’ homoeopathy in its own, allopathic, terms, a process that took around 50 years to accomplish.

By contrast, homoeopaths in the latter half of the 19th century failed to draw upon the principle of the reverse action of drugs in the way that Hahnemann had done, so effectively and comprehensively, earlier in the century. Homoeopaths’ overly empiricist stance, which contributed to their handling experimental error ineffectively, facilitated a categorical rather than a continuum-based explanatory framework (Brierley-Jones, 2005). This undermined any potentially effective nihilative strategy. Whilst homoeopaths did allude to the allopathic use of small doses and, later in the century, even pointed to instances of allopathic use of the principle of *similia*, homoeopaths failed to explain the therapeutic action of *material doses* within the framework of *their own* symbolic universe. That is, they did not characterise allopathic practice as a logical component of their own philosophy. The reason for homoeopathy’s inability to make the reverse action of drugs their own is undoubtedly related to their social organisation (Bloor, 1978; Brierley-Jones, 2005; Douglas, 1996), and could be the subject of further research.

Furthermore, homoeopathy in the first half of the 19th century had consisted of ‘converts’ from allopathic medicine, those who had come to homoeopathy through direct and often ‘miraculous’ curative experiences. By contrast, the latter half of the century was characterised by homoeopaths who had come to the practice through education. They lacked the former’s zeal for homoeopathic ‘truth’. Their different stances towards allopathic medicine can be seen as the result, in part, of the relative ease of professional socialisation and the legitimation of knowledge via direct experience, characteristic of early 19th-century ‘converts’ to homoeopathy, compared to those mediated through education in the latter half of the century, which required more effective socialisation processes and symbolic legitimation (Berger and Luckmann, 1991[1966]).

The role of language was pivotal in the allopathic nihilation of homoeopathy and in maintaining the integrity of allopaths’ medical identity. Language provides a means whereby knowledge creation, or in this case translation, can be made possible (Suzuki, 1995). This is in part because, as has been argued here, medical language is underdetermined by both medical theory and practice (Harding, 1976). That is, the same knowledge, concepts, and practices can be linguistically represented in different ways, suggesting that divergent medical systems may be commensurable and that it is medical language, more so than medical practice (Warner, 1997), that underpins medical identity.

There is a sense in which, as a loser of history, homoeopathy cannot win at the hands of historians. In its attempts to gain scientificity, 19th-century homoeopathy is deemed dilute (Haller, 2005, 2009), inauthentic, and bastard (Nicholls, 1988), whereas attempts at ‘purity’ are characterised as religious, esoteric, and sectarian (Albanese, 1986; Haller, 2005, 2009; Warner, 1997). In this article, the boundaries supposedly separating homoeopaths from each other and homoeopaths from allopaths have been either challenged or drawn differently. As a result, homoeopathy’s ‘otherness’ is reduced. Rothstein’s (1985[1972]) and Whorton’s (2002) accounts are exceptions in that they significantly reduce this ‘otherness’, with the latter admitting, ‘Unquestionably some reported [homoeopathic] cures were genuine’ and ‘there may well be more things in heaven and earth than have hitherto been dreamt of in mainstream medical philosophy’ (ibid.: 23). It is argued here that such an ‘open’ stance to homoeopathy facilitates a fuller recovery of its, and therefore medicine’s, history because it enables us to bracket what is, at best, Whiggish presentism, and at worst, reflected allopathic persecution (Haller, 2005, 2009) resulting from the ‘hatred for homoeopathy, “that common sewer of the profession”’ (Whorton, 2002: 73).

A Tory historiographical perspective not only recovers a fuller account of medical history but, as a future-oriented response, suggests what might be valuable and worth recovering and preserving from any abandoned trajectory. It encourages a critical stance to the received view, which then provides the conceptual space to consider a meta level at which homoeopathy and allopathy (biomedicine) might be commensurable. In this article, an historical (and potential future) candidate has been suggested: the principle of the reverse action of drugs. Such a Hegelian synthesis overcomes the obvious philosophical incommensurability of *similia similibus curentur* (using similars) with dilute drugs and *contraria contrariis curentur* (using opposites) with material drugs by arguing that the ‘observations of both are translated as complementary parts of a systematic understanding of reality’ (Fuller, 2002: 407). Defenders of scientific medicine will no doubt object to the absence of a known modus operandi for homoeopathy, but similar problems failed, historically, to retard development in other fields (e.g. quantum mechanics: ibid.), as has biomedicine’s lack of known mechanisms for some widely used conventional drugs (Johnson, 2015), not all of which have proven efficacy (McGoey, 2010). In an age of complex, chronic disease where care often has to substitute for cure, we might do well to keep an open mind on the future of medicine and consider whether, in terms of fully understanding drug action on the human body, homoeopathy’s history offers insight into what medicine might still be missing.

## Notes

1. The LHH treated 59,128 patients in 1886 alone.

2. Homoeopaths also ran a well-known ophthalmic hospital in New York City.

3. Homoeopaths had been banned from professional association with the AMA and the British Medical Association since the mid 19th century due to the ‘consultation clause’ written into the constitution of each society.

4. Sources were accessed at the Wellcome Library, London, UK; the Glasgow Homoeopathic Library, Glasgow, UK; the Robert Bosch Institute Library, Stuttgart, Germany; and the former Northern College of Homoeopathic Medicine, Gateshead, UK.

5. Burnett also developed and experimented with a cancer nosode derived from a breast carcinoma.
